# Bone recurrence after radical hysterectomy and lymphadenectomy in early-stage cervical cancer

**DOI:** 10.4274/tjod.galenos.2019.26932

**Published:** 2020-02-28

**Authors:** Caner Çakır, Dilek Yüksel, Çiğdem Kılıç, Mehmet Ünsal, Rıza Dur, Gökhan Boyraz, Alper Karalok, Özlem Moraloğlu Tekin, Taner Turan

**Affiliations:** 1 Etlik Zübeyde Hanım Women’s Health Training and Research Hospital, Clinic of Gynecologic Oncology, Ankara, Turkey

**Keywords:** Bone recurrence, uterine cervical cancer, survival, salvage therapy

## Abstract

**Objective:**

To present the clinical, surgical, and pathologic features of bone recurrence in patients who underwent radical hysterectomy for early-stage uterine cervical cancer.

**Materials and Methods:**

Data of 412 patients who underwent type III radical hysterectomy and pelvic ± paraaortic lymphadenectomy for stage 1B-2A epithelial cervical cancer were reviewed. Seven (1.7%) patients with bone recurrence in the first recurrence were included in the study.

**Results:**

The median follow-up of the main cohort (n=412) was 46 (range=1-300) months. In this period, recurrence developed in 53 (12.9%) patients and recurrence was observed in bone in 13.2% (7 of 53) of these recurrences. Time to recurrence ranged from 9 to 45 months. Of the recurrences, five were in the axial skeleton and two were in the appendicular skeleton. Recurrence was observed in lumbar vertebrae in three patients, thoracic vertebrae in one patient, sacral vertebrae in one patient, lumbosacral vertebrae in one patient, and the left femur in two patients. Four patients had multiple recurrence in 3 patients despite isolated bone recurrence. Patients with multiple recurrences died within 6-25 months. All isolated bone recurrences were in the axial skeleton. Complete clinical response with salvage therapy was achieved in two patients with isolated bone recurrence.

**Conclusion:**

Complete clinical response and long postoperative survival can be achieved with salvage treatment when bone recurrence is solitary in cervical cancers.

**PRECIS:** To identify the possible risk factors for postpartum urinary retention.

## Introduction

Uterine cervical cancer (CC) is the third most common cause of cancer having the highest mortality rate in the female reproductive system^(1)^. Prognostic factors of CC are based on stage, patient age, type and size of tumor, lymph node metastases, parametrial invasion, and lymphovascular space invasion^(2,3)^. Mostly, recurrence occurs within 2 years after primary treatment and 90% of patients with recurrence die^(4,5)^. The 10-year recurrence rate is reported as 3% for stage IA, 16% for stage IB, 31% for stage IIA, 26% for stage IIB, 39% for stage III, and 75% for stage IVA^(6)^.

Just like other solid tumors, CC spreads through direct invasion, and lymphatic and hematogenous dissemination. Distant metastasis to other organs such as lung, bone, braini and liver uses the hematogenous route primarily^(7,8)^. Distant organ metastasis is most commonly seen in lungs (21%), bone (16%), para aortic nodes (11%), the intestinal space (8%), and supraclavicular lymph nodes (7%). The number and site of metastasis are important for survival. The median survival is 24 weeks in lymphatic recurrence, whereas it is only 12 weeks in metastasis to other organs^(6)^. Patients with stage I-IIA CC who undergo surgery have bone recurrence in the first 2 years of the postoperative period, and usually the recurrence occurs in axial bone, especially in the vertebra^(9,10)^. The median survival after bone recurrence is reported as between 5 and 12 months^(9,10,11,12)^. In this research, our aim was to evaluate the clinical, surgical, and pathologic factors in patients with bone recurrence after type III radical hysterectomy with pelvic ± para aortic lymphadenectomy for CC.

## Materials and Methods

Between 1993 and 2018, 412 patients with stage IB-IIA epithelial CC as classified by the International Federation of Obstetricians and Gynecologists (2014) were treated with laparotomy and type III radical hysterectomy with pelvic ± paraaortic lymphadenectomy, and their data were reviewed. Seven (1.7%) patients who had the first recurrence in bone were included. Data of the patients’ clinical findings, site of recurrence, time to recurrence, treatment modality, and the response rates were obtained from the patient files and pathology reports in our gynecologic oncology clinic electronic database system.

Bone scintigraphy, magnetic resonance imaging, and positron emission tomography-computed tomography (PET-CT) were used in the diagnosis of metastatic lesions. For the differential diagnosis of metastasis, systemic examination, chest X-ray, abdominopelvic and thoracic CT and PET-CT were performed. Recurrence that developed only in bone was classified as “isolated recurrence” and bone and other sites were classified as “multiple recurrences”. Recurrence of the bone was classified as “axial skeleton”, which included cranium, sternum, vertebra and costa, and “appendicular skeleton”, which included the upper and lower extremities, scapula, and the pelvic bones. The size of the tumor was defined by the largest diameter of the tumor in the cervix at the initial treatment.

The plan of treatment in the patients with recurrence was decided by the council of gynecology and oncology. Treatment results were evaluated according to the guidelines of the World Health Organization. We defined the clinical response as follows: (I) complete clinical response: disappearance of the macroscopic tumor; (II) partial clinical response: shrinkage over 50% of the macroscopic tumor, (III) stable disease: macroscopic shrinkage of the tumor less than 50% or not less than 25% growth; (IV) progressive disease: more than 25% growth of the macroscopic tumor or macroscopic appearance of new tumor foci^(13)^.

The factors indicating the bone recurrence could not be recognized at this point, because the number of the patients with bone recurrence were only 7 (1.7%). The time of from surgery until recurrence was defined as time-to-recurrence (TTR), the time until the death of the patient was defined as overall survival, and the time of recurrence until the death of patient or until the last date was defined as post recurrence survival.

All patients were followed up after the initial treatment for the CC. Patients who had complete clinical response with salvage treatment for recurrence were followed up quarterly in the first 2 years, semi-annually for up to 5 years, and annually thereafter. Pelvic examination, abdominopelvic ultrasonography, Papanicolaou smear, complete blood count, and biochemistry profile were performed in the follow-up. Chest X-ray was used annually unless there was clinical suspicion. Thoracic and\or abdominal CT were used when needed.

## Results

The median follow-up of the main cohort (n=412) was 46 (range=1-300) months. In this period, recurrence developed in 53 (12.9%) patients, and the recurrence rate in bone was observed as 13.2% (7 of 53). Tumor type was squamous carcinoma in six patients and mixed type in one patient (squamous + adenocarcinoma). Paraaortic lymphadenectomy was added to the surgical procedures in six patients and pelvic lymphadenectomy alone was performed in one patient. The number of lymph nodes removed was between 42 and 102. It was determined that there was spread to the pelvic lymph nodes in two patients and pelvic and paraaortic lymph nodes in one patient. There was parametrial invasion in one patient, surgical border positivity in one, and lymphovascular space invasion in two patients. The surgical-pathologic features are shown in Table 1.

One patient (patient #7) received neo-adjuvant chemotherapy. As neoadjuvant chemotherapy, the patient received a combination of cisplatin + tegafur-uracil for 2 cycles. Adjuvant therapy was given to three patients as concurrent chemo-radiotherapy (CCRT) and three patients received no adjuvant therapy. One patient (patient #4) refused adjuvant therapy.

TTR ranged from 9 to 45 months. Five of the recurrences were in the axial skeleton and two were in the appendicular skeleton. Recurrence was observed in three patients in the lumbar vertebrae, one in the thoracic vertebrae, one in the sacral vertebrae, one in the lumbosacral vertebrae, and two in the left femur. Three patients (patient #1, #6, and #7) had isolated bone recurrence and four patients had multiple recurrence. Except for the bone, one of them had it in the inguinal and supraclavicular lymph nodes, one in pelvic-paraaortic lymph nodes, one in lung and paraaortic lymph nodes and one in lung (Table 2). Recurrence was in the axial skeleton in all isolated bone recurrences.

After recurrence, six patients received salvage therapy for curative intent and one patient received palliative therapy (patient #4). Two of the patients who received salvage therapy were given only systemic treatment (cisplatin + 5 fluorouracil). Four patients received radiotherapy, two of whom were given systemic treatment after radiotherapy. Radiotherapy was performed in one patient with weekly cisplatin (CCRT) and one patient received radiotherapy only (Table 2). In salvage therapy, one patient with only systemic treatment and one patient with radiotherapy had a complete clinical response (patients #1 and #7). These two patients had isolated bone recurrence and their post recurrence survival was 129 months and 11 months, respectively. During the follow-up period, four patients died because of the disease (patient #2, #3, #4 and #5). The recurrence type of these four patients was multiple recurrence, and in two the disease recurred in the lung (Table 2). These patients died within 6-25 months after recurrence. Recurrence was seen in the femur and pelvic paraaortic lymph nodes of the patient who lived up to 25 months after recurrence and was treated with concurrent chemo-radiotherapy.

## Discussion

The results of bone recurrence in uterine CC vary widely. Drescher^(14)^ reported bone metastasis in 1.2%. However, this rate was 16% in the study of Fagundes et al.^(6)^. In our case series, the rate of bone recurrence was 1.3% in the main cohort where the median follow-up was 46 months.

The site and number of recurrences are the main factors affecting prognosis and treatment^(15)^. It is known that the success of treatment is low when recurrence occurs in others site accompanying the bone recurrence^(16)^. In the current case series, all patients with multiple recurrences died of recurrent disease. The choice of treatment for recurrent disease is primarily dependent on previous treatment and should be evaluated together with the location of the recurrent tumor and the patient’s performance^(17,18)^. In patients with CC who have distant and multiple recurrent disease, the primary aim of treatment is mostly not-curative intent but palliative^(19)^. However, in a study presented by Makino et al*.*^(20)^of 75 patients with uterine CC and bone recurrence, the overall survival (OS) of 16 patients who received chemotherapy and CCRT after RT was 18 months and 2 months, respectively, compared with 25 patients receiving palliative treatment (p<0.05). In our case series, complete clinical response was obtained with salvage treatment in two patients in the presence of isolated recurrence. Salvage treatment was applied to one of them with systemic treatment, and with cisplatin and radiotherapy to the other. In recurrent CC, cisplatin is preferred for most patients. Systemic treatment success rate is 12-22% in recurrent CC^(21,22,23,24,25,26)^. Unlike other anti-angiogenic agents, bevacizumab has been used as a part of cisplatin-based combination therapy for recurrent, persistent or metastatic CC, and its addition to the cisplatin-paclitaxel protocol has been shown to increase OS from 13 months to 17 months (p=0.008)^(27,28)^. Surgical treatment has been applied in selected cases with solitary bone recurrence in the literature. Ida et al*.*^(29)^ were able to control the disease by surgical resection in a solitary femur recurrence that developed 22 months after the first treatment. However, Makino et al*.*^(20)^ reported that in two patients with solitary bone recurrence, complete resection could not be achieved. We had no patients who could be managed surgically in this series.

The retrospective nature is the main limitation of the study. The number of patients was low and this prevented clear results to change clinical practice. However, the study evaluated patients who had a median follow-up of approximately 4 years and who had undergone radical surgery from among more than 400 early-stage cancers.

## Conclusion

In conclusion, complete clinical response and long postoperative survival can be achieved with salvage treatment when bone recurrence is solitary. However, the effect of surgery in this patient group should be evaluated. Multimodal treatment options including surgery in CC with bone recurrence, especially solitary recurrence, need to be evaluated in further studies.

## Figures and Tables

**Table 1:**
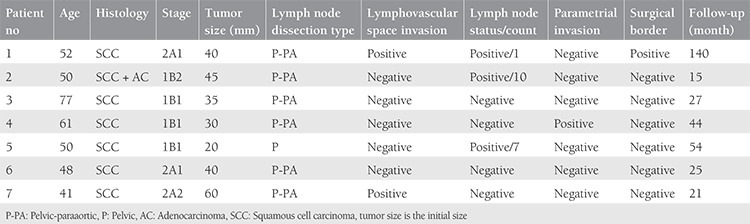
Clinical, pathologic, and surgical features

**Table 2:**
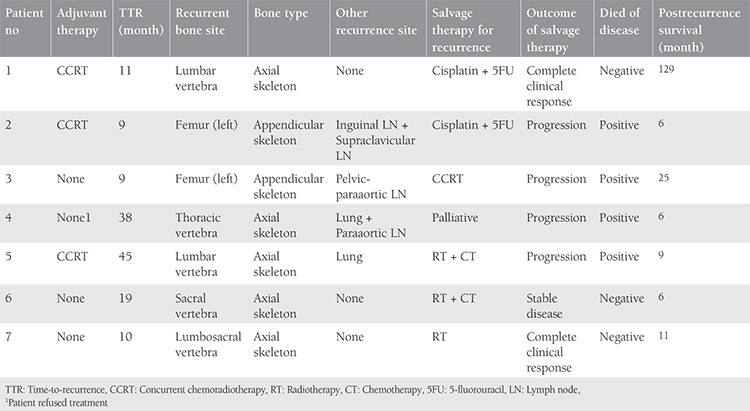
Bone metastasis site, treatment and outcome
